# Purification of family B G protein-coupled receptors using nanodiscs: Application to human glucagon-like peptide-1 receptor

**DOI:** 10.1371/journal.pone.0179568

**Published:** 2017-06-13

**Authors:** Yingying Cai, Yuting Liu, Kelly J. Culhane, Brian T. DeVree, Yang Yang, Roger K. Sunahara, Elsa C. Y. Yan

**Affiliations:** 1 Department of Chemistry, Yale University, New Haven, Connecticut, United States of America; 2 Department of Molecular Biophysics and Biochemistry, Yale University, New Haven, Connecticut, United States of America; 3 Department of Chemistry, University of Michigan, Ann Arbor, Michigan, United States of America; 4 Nanobiology Institute, Yale University, New Haven, Connecticut, United States of America; 5 Department of Cell Biology, Yale University School of Medicine, New Haven, Connecticut, United States of America; 6 Department of Pharmacology, University of California at San Diego, La Jolla, California, United States of America; Indian Institute of Technology Kanpur, INDIA

## Abstract

Family B G protein-coupled receptors (GPCRs) play vital roles in hormone-regulated homeostasis. They are drug targets for metabolic diseases, including type 2 diabetes and osteoporosis. Despite their importance, the signaling mechanisms for family B GPCRs at the molecular level remain largely unexplored due to the challenges in purification of functional receptors in sufficient amount for biophysical characterization. Here, we purified the family B GPCR human glucagon-like peptide-1 (GLP-1) receptor (GLP1R), whose agonists, e.g. exendin-4, are used for the treatment of type 2 diabetes mellitus. The receptor was expressed in HEK293S *GnTl*^-^ cells using our recently developed protocol. The protocol incorporates the receptor into the native-like lipid environment of reconstituted high density lipoprotein (rHDL) particles, also known as nanodiscs, immediately after the membrane solubilization step followed by chromatographic purification, minimizing detergent contact with the target receptor to reduce denaturation and prolonging stabilization of receptor in lipid bilayers without extra steps of reconstitution. This method yielded purified GLP1R in nanodiscs that could bind to GLP-1 and exendin-4 and activate G_s_ protein. This nanodisc purification method can potentially be a general strategy to routinely obtain purified family B GPCRs in the 10s of microgram amounts useful for spectroscopic analysis of receptor functions and activation mechanisms.

## Introduction

G protein-coupled receptors (GPCRs) constitute the largest family of membrane proteins that detect extracellular stimuli and activate intracellular signal transduction pathways. All GPCRs share a common seven-transmembrane topology, and are often classified into five main sub-families (A-E) [1] based on their functions and sequence similarities [2, 3]. Since ligand-binding sites of GPCRs are highly specific and receptor activation regulates almost all physiological processes, GPCRs have been heavily studied and exploited as drug targets [4–9].

**Fig 1:**
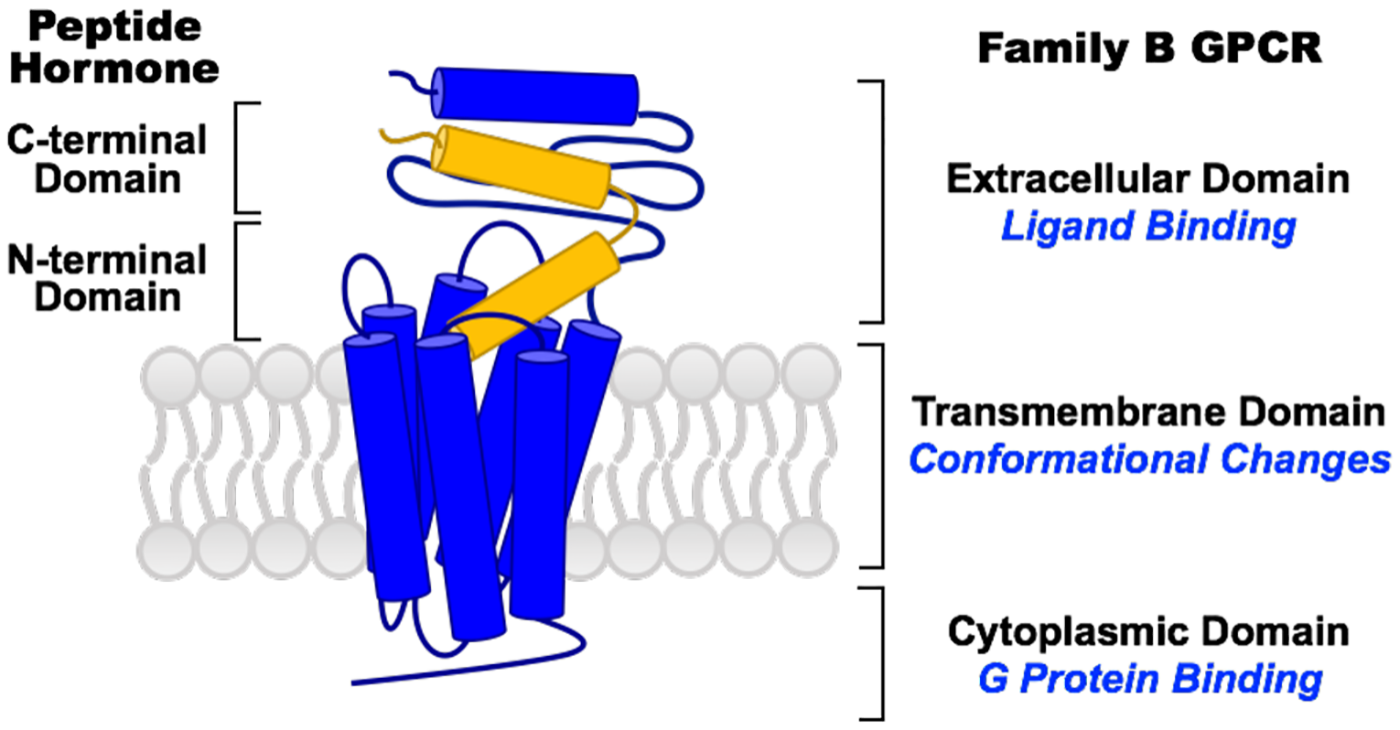
Scheme of a peptide hormone (yellow) bound family B GPCR (blue) in lipid bilayer (grey).

Recently, family B GPCRs, a relatively small family with 15 members, have gained increasing attention for the treatment of metabolic diseases, such as osteoporosis and type 2 diabetes [9, 10]. Family B GPCRs have relatively large N-terminal extracellular domains that share a similar fold for ligand binding (Fig 1). They use peptide hormones as their native ligands [11]. Ligand binding causes conformational rearrangements, propagating from the transmembrane region to the cytoplasmic domain, which trigger downstream signaling cascades via G protein coupling (Fig 1) [12]. Understanding the signaling mechanisms of family B GPCRs is of great importance in cellular signaling processes and drug development.

As a member of family B GPCR, glucagon-like peptide-1 receptor (GLP1R) is highly expressed in pancreatic beta cells. It couples to G_q_ and G_i_ proteins that regulate cellular level of calcium and diacylglycerol, respectively. In addition, it couples to the stimulator G protein, G_s,_ that activates adenylyl cyclase and raises intracellular cyclic AMP (cAMP), a predominant signaling pathway triggering the synthesis and release of insulin and thereby lowering glucose concentrations [13, 14]. Thus, GLP1R is one of the best-validated drug targets for type 2 diabetes [15, 16]. The G protein coupling of GLP1R is triggered by ligand binding. Its cognate ligand is glucagon-like peptide 1 (GLP-1), an incretin hormone secreted after food consumption to facilitate glucose disposal from intestinal endocrine cells. The hormone is secreted in 2 major forms: GLP-1-(7–37) and GLP-1-(7–36)-NH_2_ [17–20]. GLP-1 and its analogues are therefore a subject of intensive investigation for type 2 diabetes treatments, and the focus has been on improving their metabolic properties. For instance, exendin-4 (Ex-4) is a 39-amino acid agonist of GLP1R and its synthetic version (exenatide) is an FDA-approved drug. A molecular-level understanding of function and activation mechanism of GLP1R can aid rational design of drugs targeting GLP1R for diabetes treatment.

A fundamental understanding of signaling mechanism of GLP1R and other family B GPCRs requires not only knowledge about their static structures but also information about the dynamics of structural changes during the signaling process. Although the structures of the ligand-bound N-terminal domain as well as truncated transmembrane domain of two family B GPCRs have recently been reported [21–25], how the ligand binding domain and the transmembrane domains come together to form the full-length receptor and how they work synergistically to transduce signal across cell membrane remain largely unexplored [26, 27]. One major challenge is to purify sufficient amount of full-length functional receptors for characterizations using biophysical methods. Thus, more efficient methods for purification of family B GPCRs are needed.

**Fig 2:**
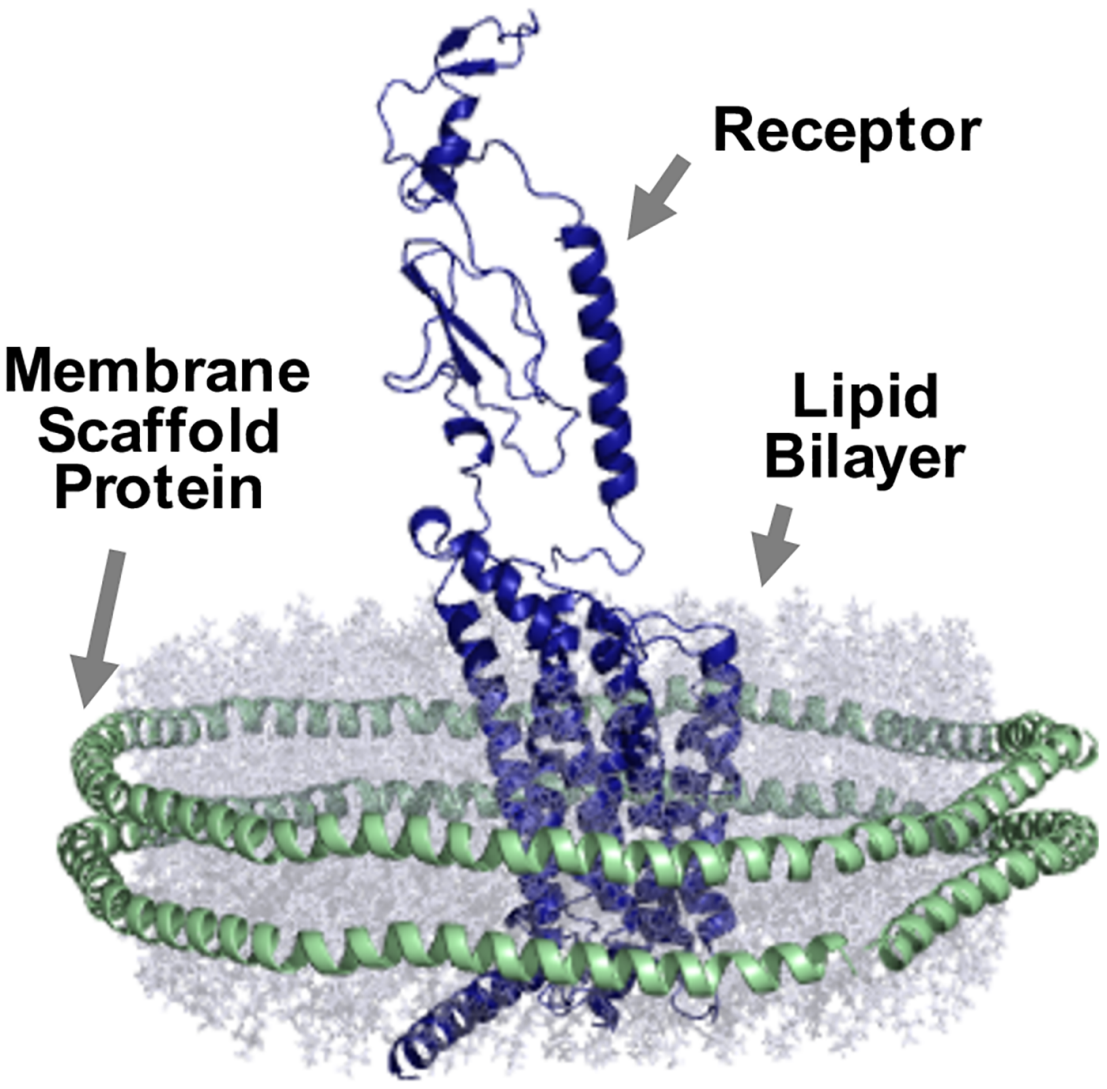
Scheme of a receptor (blue) incorporated in a nanodisc. A nanodisc is a lipid bilayer (grey) surrounded by two membrane scaffold proteins (green).

**Fig 3:**
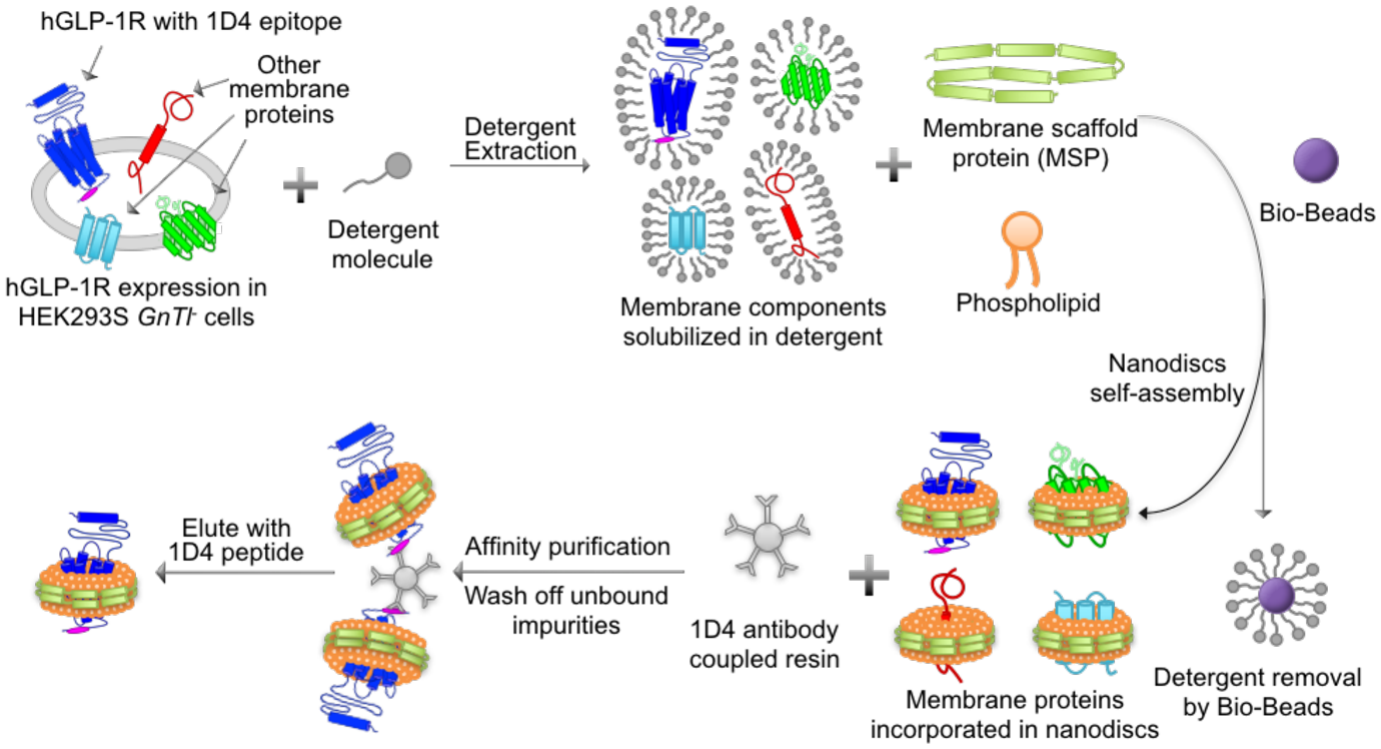
The purification method of GLP1R using nanodiscs. GLP1R tagged with the 1D4 epitope is expressed in a mammalian expression system; membrane fractions are isolated and then solubilized using detergent; solubilized membrane fractions are incubated with phospholipid and membrane scaffold proteins (MSPs); Bio-Beads are added to remove detergents and to initiate nanodisc assembly; GLP1R incorporated in nanodiscs are bound to antibody-conjugated resin and unbound nanodiscs or non-GLP1R components are washed off; 1D4 peptide was used to elute purified GLP1R-ND.

Here, we report the purification of human GLP1R expressed in mammalian HEK293 cells using nanodiscs. Nanodiscs [28, 29], also known as discoidal reconstituted high density lipoprotein (rHDL) [30] and nanoscale apolipoprotein-bound bilayer [31] (NABB), consist of a phospholipid bilayer held together by two molecules of membrane scaffold protein (MSP), [30, 32] (Fig 2). MSPs are amphipathic helical repeat proteins based on apolipoprotein A1, that wraps around the hydrophobic edge of the lipid disc to stabilize it in an aqueous environment [33, 34]. The transmembrane proteins are incorporated into the lipid bilayers of the nanodiscs immediately after membrane solubilization to minimize detergent contact, which provides structural stability (Fig 3). We have previously developed this method and successfully applied it to purify family B GPCR parathyroid hormone 1 receptor (PTH1R) [35]. Here, we report the extension of such method to human GLP1R, showing that the purified receptors in nanodiscs can bind to its native ligand and activate G_s_ in response to ligand binding. Our work represents the first functional expression and purification of full-length GLP1R from a mammalian system, providing an alternative approach of sample preparation for future biophysical studies of GLP1R in a native-like lipid environment. This study also implies potential applications of the purification method to other family B GPCRs and more generally to other transmembrane proteins.

## Material and methods

### Materials

Peptide 1D4 (TETSQVAPA) was synthesized at the Keck Biotechnology Resource Laboratory at Yale University. Two fluorescently labeled peptides, GLP-1-(7–37) and Ex-4, were also obtained from there with the E21K and L21K mutations, respectively for lysine conjugation with 5(6)-carboxyfluorescein (FAM). The following materials were purchased from indicated sources: peptide GLP-1-(7–37) from GL Biochem (Shanghai) Ltd; Ex-4 from Abcam; BODIPY-FL-GTPγS from Invitrogen; 1-palmitoyl-2-oleoyl-sn-glycero-3-phosphocholine (POPC) from Avanti Polar Lipids; n-dodecyl-β-D-maltopyranoside (DDM) from Anatrace; Bio-Beads SM-2 from Bio-Rad; and Rho 1D4 purified monoclonal antibody from University of British Columbia. The antibody was coupled to UltraLink Hydrazide resin purchased from Thermo Scientific [36, 37]. All other chemicals were analytical grade obtained from Sigma or American Biochemicals. Membrane scaffold protein (MSP1E3D1) was expressed and purified as described previously [33, 35].

### Construction of the GLP1R plasmid

GLP1R clone was obtained from Open Biosystems and site directed mutagenesis was applied to remove the embedded *Kpn*I site. Using PCR, restriction sites *Kpn*I and *Not*I were introduced at the 5’ and 3’ end respectively, as well as a 1D4 epitope, recognized by the 1D4 antibody, immediately before the stop codon at the 3’ end. The GLP1R cDNA was then subcloned at *Kpn*I and *Not*I sites into the tetracycline inducible pACMV-tetO vector [38, 39]. The accuracy of the construct was confirmed by sequencing the entire plasmid (Keck Biotechnology Resource Laboratory, Yale University).

### Generation of HEK293S stable cell line for GLP1R expression

HEK293S *GnTl*^-^ cells [38, 39] were maintained in 1:1 Dulbecco’s modified Eagle’s medium/Ham's F12 Nutrient Mixture (DMEM/F12), supplemented with 10% of fetal bovine serum (FBS) at 37°C in a humidified atmosphere containing 95% air and 5% CO_2_. At ~80% confluency, transfection of HEK293S cells was performed using Lipofectamine Plus^™^ transfection reagent with purified plasmid containing the GLP1R cDNA, following the established protocol. After transfection, cells were allowed to grow for geneticin resistance under non-selective conditions for at least 24 hours, and then treated with 0.5 mg/ml geneticin for about 3 weeks until outgrowth of resistant cells. The established stable cell line (un-induced) was frozen down with 10% DMSO at -80°C for storage. The receptor expression level was evaluated using western blot after inducing the cells with 0.55 mg/mL sodium butyrate and 2 μg/mL tetracycline and maintained for ~40h.

### Isolation of membrane fractions

The GLP1R stable cells were grown in 10-cm tissue-culture dishes to confluence of 80%, induced with 0.55 mg/mL sodium butyrate and 2 μg/mL tetracycline, and maintained for another 40h under 5% CO_2_/ 95% air in 1:1 DMEM/F12 supplemented with 10% FBS for over expression of GLP1R. Induced cells were washed with phosphate-buffered saline and harvested in hypotonic buffer (10 mM Tris pH 7.4 and 4 mM EDTA) with Complete Protease Inhibitor (Roche). Then, the cells were lysed and homogenized by passing through a 26 gauge syringe. The homogenates were centrifuged at 1500 g for 10 min at 4°C. The resulting pellets were resuspended in 1.5 mL of solution A (0.25 M sucrose, 10 mM Tris pH 7.4, and 1 mM EDTA) containing Complete Protease Inhibitor, then mixed thoroughly with 2X volumes of solution B (2 M sucrose, 10 mM Tris-HCl, pH 7.4, and 1 mM EDTA) with Complete Protease Inhibitor. The mixture was layered with solution A (1/10X mixture volume) and centrifuged at 113,000 g for 30 min. The membrane enriched pellets were collected at the interface between the two sucrose solutions and then resuspended in hypotonic buffer. The suspension was centrifuged again at 113,000 g for 15 min resulting in the membrane pellets.

### Nanodisc purification of GLP1R

As described previously [35], POPC in chloroform was dried with Argon, and re-solubilized with n-dodecyl β-D-maltoside (DDM) buffer (180 mM DDM, 20 mM Tris-HCl, pH 7.4, 100 mM NaCl, 0.5 mM EDTA); MSP1E3D1 was expressed in *E*.*coli* carrying a His-tag and purified with Ni-NTA matrix; and the membrane fraction of GLP1R expressed HEK293 cells was isolated using sucrose density gradient ultracentrifugation as described above. As illustrated in Fig 3, the membrane pellets from the cells grown in 30–60 plates were solubilized in the solubilization buffer (50 mM Tris-HCl, pH 7.4, 150 mM NaCl, 5 mM CaCl_2_, 5 mM MgCl_2_, 2 mM EDTA, 10% glycerol, 0.5% DDM). The total protein concentration of the solubilized membranes was quantified using the DC protein assay (Bio-Rad) and 40,000 as an average molecular weight [40]. A mixture of 11 μM of total membrane protein, 90 μM MSP1E3D1 and 8 mM POPC was prepared in a buffer of 50 mM Tris-HCl pH 7.4, 150 mM NaCl, 5 mM MgCl_2_, 5 mM CaCl_2_, 4 mM EDTA, and 4% glycerol. The mixture was incubated on ice for 30 min and aliquoted 400 μL each into 1.5 mL centrifuge tubes containing ~0.3 mL of Bio-Beads. The mixture with Bio-Beads was then gently rotated at 4°C overnight. Upon removal of detergent by Bio-Beads, nanodiscs spontaneously self-assembled, incorporating all the membrane components from the expression system. After the Bio-Beads was removed via centrifugation, resin containing immobilized Rho 1D4 monoclonal antibody were added to the mixture to specifically bind to the 1D4 epitope tag on GLP1R. The resin was then washed and the bound GLP1R incorporated in ND (GLP1R-ND) was eluted with 1D4 peptide (0.37 mg/ml) in the buffer of 50 mM Tris-HCl pH 7.4, 150 mM NaCl, and 3 mM MgCl_2_ to yield the purified receptor.

### Dynamic light scattering

Size distribution of GLP1R-ND was measured by dynamic light scattering (DLS) at 4°C using a DynoPro Plate Reader II (Wyatt Technology) equipped with a 75 mW linearly polarized diode laser as the light source with wavelength at 830 nm and scattering angle at 158°. Samples were prepared in the buffer of 50 mM Tris-HCl, pH 7.4, 150 mM NaCl, and 3 mM MgCl_2_ at a final GLP1R-ND concentration of ~0.5 mg/ml, filtered and introduced into 384-well clear flat bottom black microplate (Corning). The light scattering data were analyzed using the DYNAMICS (Wyatt Technology) software to yield mass-based size distribution of the samples.

### Transmission electron spectroscopy

To achieve negative-staining transmission electron microscopy (TEM) images of GLP1R-ND, a 5 μL droplet of GLP1R-ND solution at the concentration of ~80 nM was deposited on a glow discharged formvar/carbon coated copper grid (Electron Microscopy Sciences), incubated for 1 minute and blotted away. The grid was then briefly washed and stained for 1 minute with 5 μL of 2% (w/v) uranyl formate. Images were captured on a JEOL JEM-1400 Plus microscope (acceleration voltage: 80 keV) with a bottom-mount 4k×3k CCD camera (Advanced Microscopy Technologies).

### Ligand binding assays

**Fig 4:**
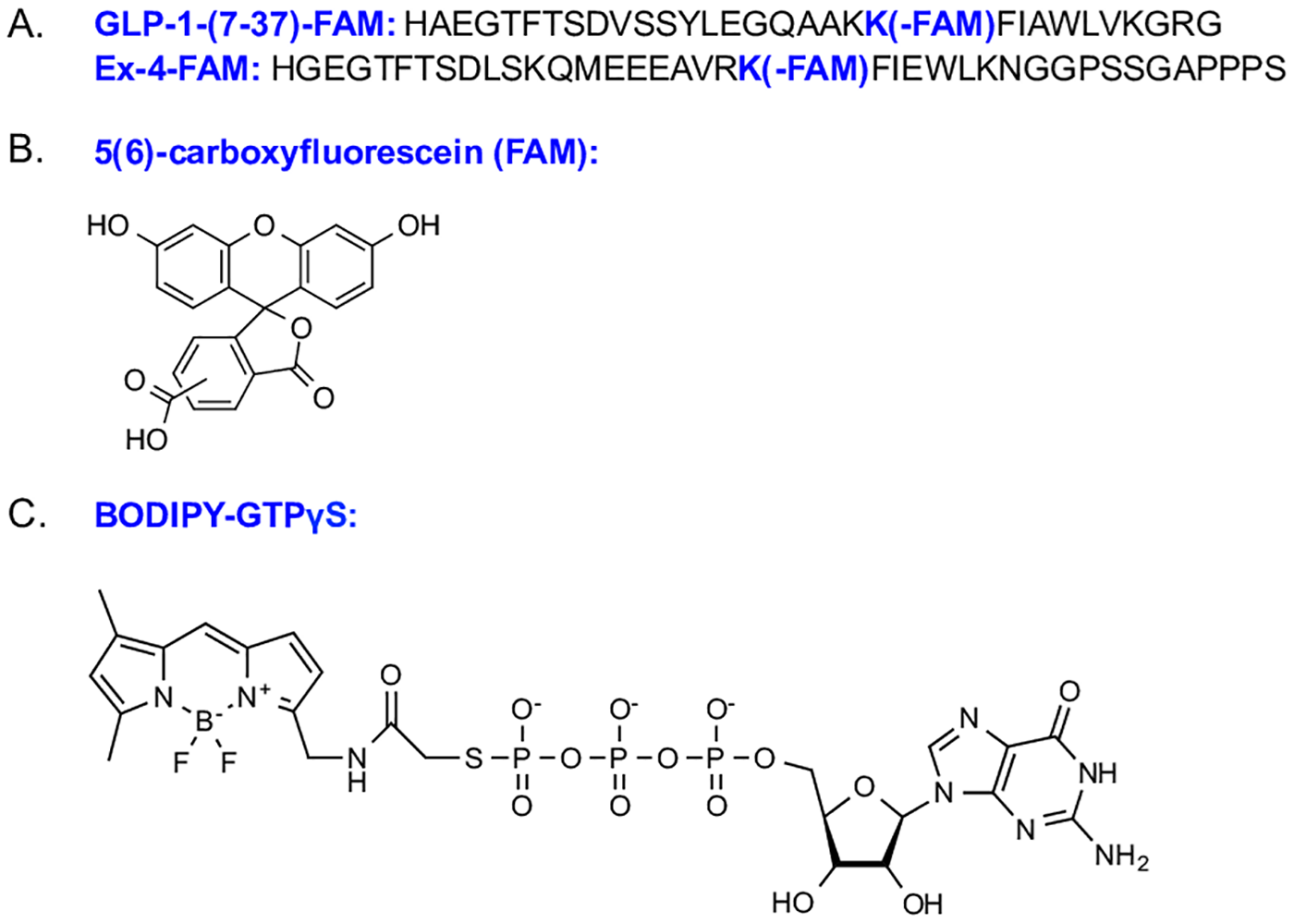
Fluorescently labeled peptides for ligand binding assays. (A) Sequences of FAM-labeled GLP-1-(7–37) and Ex-4; (B) Chemical structure of the fluorescent dye 5(6)-carboxyfluorescein (FAM); (C) Chemical structure of BODIPY-FL-GTPγS.

Fluorescence anisotropy was used to measure the binding between purified GLP1R-ND and GLP-1-(7–37) or Ex-4. GLP-1-(7–37) was labelled with 5(6)-carboxyfluorescein (FAM) (Fig 4) at the E21K site while Ex-4 was labelled with FAM at the L21K site. Crystal structures show that these labeling sites are solvent exposed [21, 22, 41]. In a cuvette, 50 nM of GLP1(17–37)-FAM or Ex-4-FAM was incubated with freshly prepared GLP1R-ND at various concentrations, ranging from 0 to 400 nM, in 20 mM Tris-HCl, pH 7.4, 150 mM NaCl, 100 M EDTA, and 3 mM MgCl_2_. All samples containing FAM labeled peptide were prepared under red dim light and kept in dark until the anisotropy measurement. The anisotropy was measured on a PTI QuantaMaster C-61 two-channel fluorescence spectrophotometer at room temperature with excitation/emission of 497 nm/518 nm both at slit widths of 5 nm. Each measurement was averaged over 30 s with a time interval of 1 s. Anisotropy data averaged from three independent measurements were analyzed and fitted to a simple single-site binding model, as described previously [35], to obtain the dissociation constant, *K*_*D*_.

### Expression and purification of G-proteins

Baculovirus-mediated expression of G_s_ heterotrimer (G_α_, G_β_, and G_γ_) in *Trichoplusia ni* (HighFive^™^; Invitrogen) insect cells was performed as previously described in Rasmussen *et al* 2011 [42]. G_s_ heterotrimer was purified by metal ion affinity chromatography (Ni-NTA), ion-exchange chromatography and size exclusion chromatography from detergent solubilized (DDM) membranes essentially as described in Rasmussen *et al* 2011[42].

### G protein activation assay

The G_s_ activation was measured based on the increase of fluorescence intensity of BODIPY-FL-GTPγS upon binding to G protein. BODIPY-FL-GTPγS (Fig 4C) is a non-hydrolyzable fluorescently labeled GTP analogue. The fluorescence of BODIPY-FL-GTPγS is 90% quenched relative to that of the BODIPY dye alone, but the quenching is partially restored upon binding to G proteins. Hence, an increase in fluorescence indicates binding of BODIPY-FL-GTPγS to G proteins, implying G protein activation. The fluorescence intensity was monitored in real time using Cary Eclipse fluorescence spectrophotometer at 30°C with excitation/emission of 500nm/511nm and the slid width of 2.5 nm and 5 nm respectively. The spectrophotometer was equipped with a multi-cell peltier block, allowing the simultaneous measurement of up to four samples. To measure the G protein activation induced by the purified receptor, a mixture of 100 nM G_s_, 100 nM BODIPY-FL-GTPγS, and 20 nM GLP1R-ND were prepared in reaction buffer (20 mM Tris-HCl, pH 7.4, 150 mM NaCl, 3 mM MgCl_2_). Immediately 54 μL of such mixture was aliquoted to each measuring cuvette, at which time the spectrophotometer started to record the fluorescence intensity. After 5 min, allowing the signal to stabilize, 6 μL agonist ligand of either GLP-1-(7–37) or Ex-4 at a stock concentration of 20 μM was added to the appropriate cuvette to yield a final peptide concentration of 2 μM. As a control, one of the four measuring cuvettes was added with 6 μL of reaction buffer, instead of the ligand. The fluorescence intensity was continuously monitored for 3 h since then. Furthermore, to verify G protein activation requires the purified receptor, another control was simultaneously performed in one of the four measuring cuvettes, where the 54 μL of reaction mixture includes only G_s_ and BODIPY-FL-GTPγS but without GLP1R-ND, and that after 5 min incubation, a 6 μL of GLP-1-(7–37) stock solution at 20 μM was added to the system.

## Results

### Expression and purification of GLP1R

**Fig 5:**
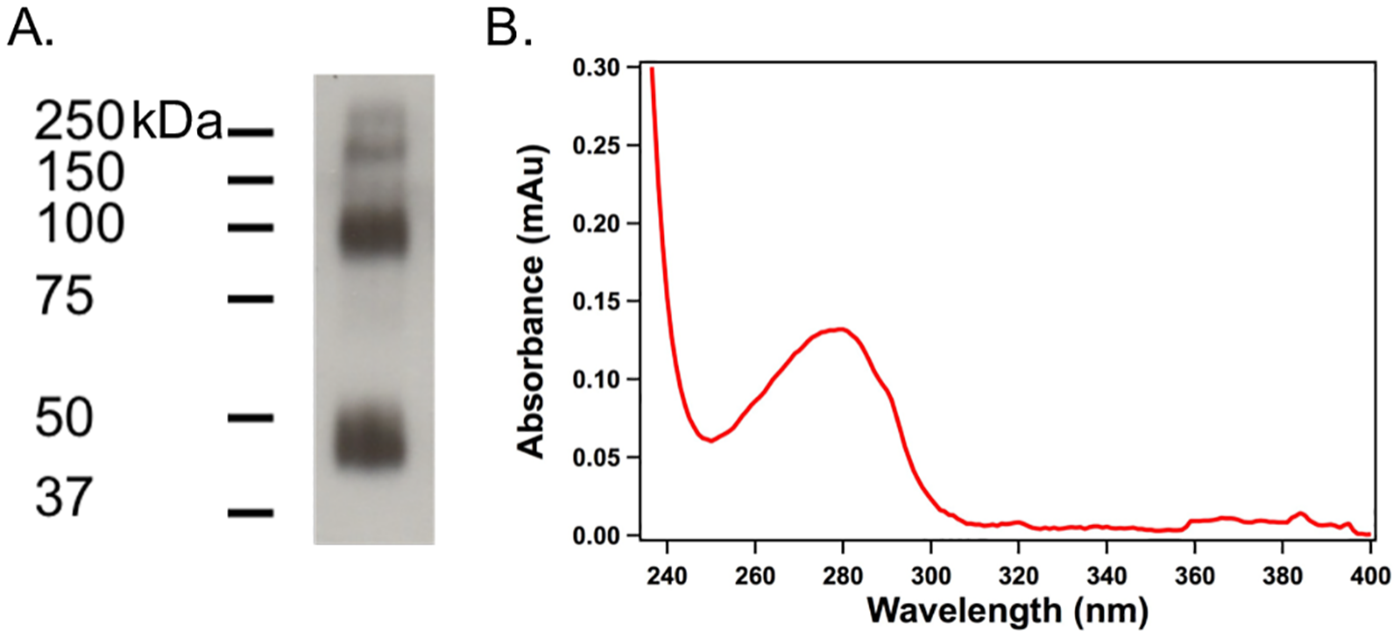
Expression and purification of GLP1R. (A) Western blot showing the transfection and expression of GLP1R in HEK293S cells. The two major SDS-resistant bands at ~50 kDa and ~100 kDa belong to the monomer and dimer states of GLP1R; (B) UV-visible spectrum of purified GLP1R in nanodiscs.

We determined GLP1R protein expression in HEK293S cells using western blot (Fig 5A). The blot performed with 1D4 antibody against 1D4 sequence tagged to GLP1R shows that the molecular weight of the major band is consistent with the monomeric GLP1R (~53 kDa). There also appears to be a band at approximately ~100 kDa representing the dimer of GLP1R [43], further indicating the expression of the receptor in the HEK293 cells stably transfected with the GLP1R gene. Such dimer resistant to SDS denaturation has previously been reported and observed in preparations with other GPCRs [44–46].

We then purified GLP1R expressed in HEK293 cells in nanodiscs. Directly after membrane solubilization with detergent, GLP1R together with other membrane proteins were incorporated into self-assembled nanodiscs formed by phospholipid (POPC) and membrane scaffold proteins (MSP1E3D1) (Fig 3). The MSP1E3D1 protein generates nanodiscs in a diameter of ~15 nm [34], which is suitable for the incorporation of GLP1R and family B GPCRs in general. To achieve one GLP1R incorporated in each disc, we optimized the nanodisc assembly protocol by determining the lipid:MSP:membrane protein ratio and the amount of Bio-Beads to be used in the nanodisc self-assembling step, as described in Mitra *et al* [35], resulting in ~ 2% of nanodiscs containing more than one membrane proteins. Further, after purification, the sample contains less than 0.01% containing two GLP1R receptors (see S1 Supporting Information).

Affinity purification with 1D4 resin yielded the final product of GLP1R in nanodiscs. Fig 5B presents the absorption spectra of purified GLP1R-NDs from 400 to 250 nm, maximum at 280 nm, from which we calculated the protein concentration with an assumption that each nanodisc consists of two molecules of MSP1E3D1 (extinction coefficient at 280 nm, ε_280_ = 29910 M^-1^·cm^-1^) and one molecule of GLP1R (ε_280_ = 125790 M^-1^·cm^-1^). Thereby, we determined the purification yield of GLP1R-ND averaged over 30 preparations to be ~0.25 μg per 10-cm tissue culture dish of HEK293 cells.

**Fig 6:**
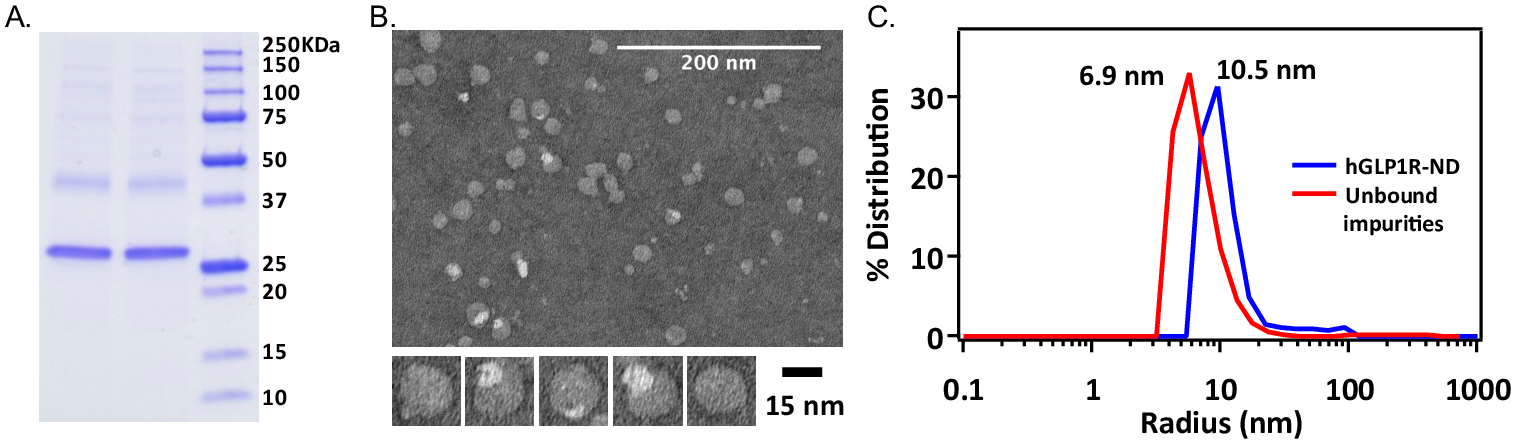
Characterization of GLP1R. (A) SDS-PAGE gel of purified GLP1R-ND, with two major bands at ~28 kDa and ~50 kDa representing MSP and GLP1R respectively; (B) Transmission electron microscopy (TEM) images of GLP1R-ND. The average size is ~18 nm; (C) Mass-based size distribution of isolated GLP1R-ND and wash-off unbound nanodiscs collected during chromatographic purification measured by dynamic light scattering (DLS).

We further characterized the purified GLP1R-NDs using SDS-PAGE, transmission electron microscopy (TEM), and dynamic light scattering (DLS). Fig 6A shows the results of SDS-PAGE analyses of GLP1R-ND with two major bands corresponding to MSP1E3D1 (~28 kDa) and GLP1R (~53 kDa) respectively. Although the GLP1R band runs smaller (~45 kDa) than its molecular weight of ~53 kDa, such phenomena of gel shifting is common among membrane proteins [47, 48]. Often, membrane protein samples are not completely denatured by SDS, thus have a more compact shape leading to faster migration in the SDS-PAGE gel, reported to be 70–85% of their expected molecular weight [49, 50]. The TEM image (Fig 6B) was analyzed with ImageJ to yield the mean particle size of 19.0 ± 1.6 nm of the nanodiscs (see S1 Supporting Information). The DLS measurement shows the average diameter of the isolated GLP1R-ND as 21.0 nm (radius of 10.5 nm, Fig 6C) and a small population of aggregates with a diameter of around 100 nm present. The calculated volume partition of aggregates in sample is less than 2% for purified GLP1R-ND. The wash-off unbound impurities collected during chromatographic purification, consisting mostly empty nanodiscs, were also examined using DLS yielding an average diameter of 13.8 nm (radius of 6.9 nm, Fig 6C). The GLP1R-ND diameter measured using DLS is larger likely due to GLP1R’s large extracellular domain that increases the hydrodynamic radius. The characterizations (Fig 6) indicated the generation of GLP1R-ND.

### Ligand binding of GLP1R-ND

**Fig 7:**
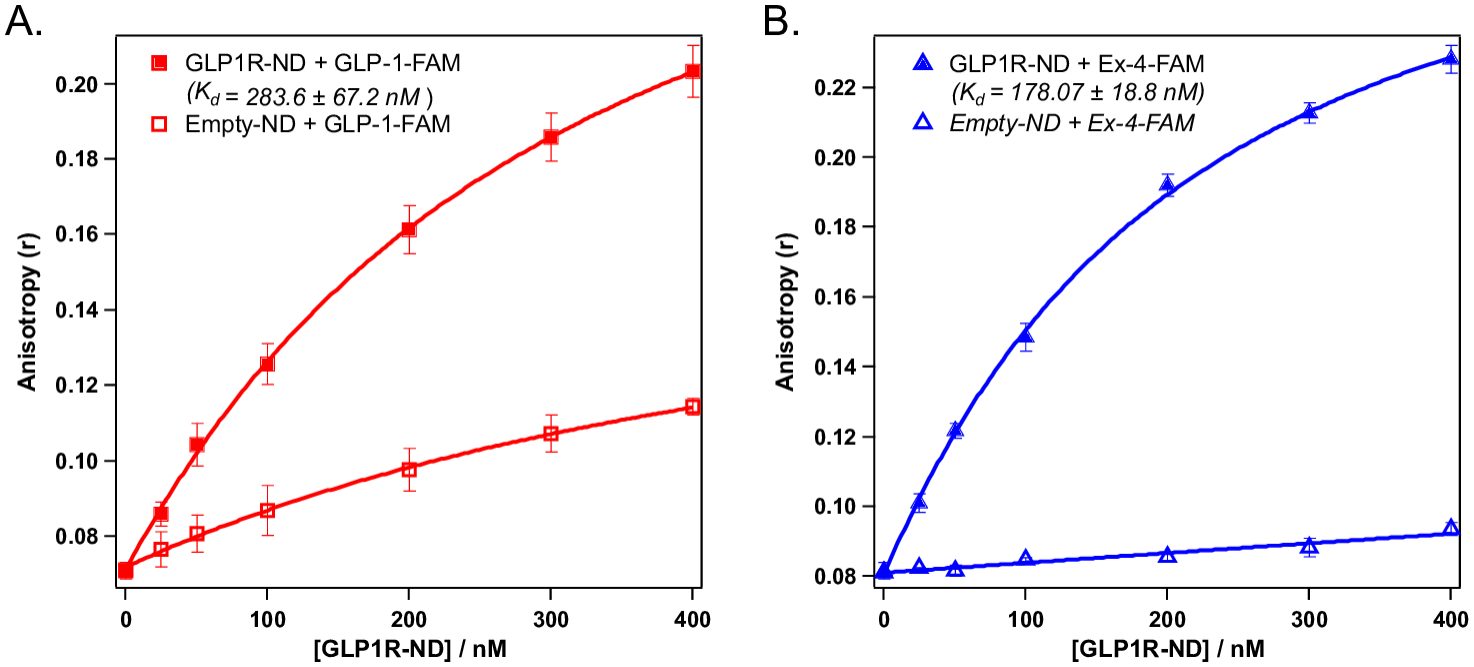
Ligand binding activity of GLP1R-ND. Titration curves of FAM labeled (A) GLP-1(7–37) and (B) Ex-4 with GLP1R incorporated in nanodiscs (GLP1R-ND) and with nanodiscs without the receptor (empty-ND). The FAM labeled peptide concentration was kept constant at 55 nM. The fluorescence was monitored at excitation/emission 497 nm/518 nm with slit widths 5 nm/5 nm. Each data point is an average of three anisotropy values measured using three distinct preparations of purified receptor with standard deviation shown as errors.

We tested the ligand binding ability of GLP1R purified in nanodiscs using fluorescence anisotropy. The fluorescently labeled peptide, GLP-1-(7–37)-FAM or Ex-4-FAM, was kept at a constant concentration of 50 nM and titrated with purified GLP1R-ND. As shown in Fig 7, the anisotropy increases with increasing concentrations of GLP1R-ND. Such an increase in anisotropy indicate the decreased tumbling rate of the fluorescent species, caused by the binding of FAM-labeled ligand to the GLP1R-ND. Titration of the ligand with empty nanodiscs showed a slight increase in anisotropy likely due to non-specific binding (see S1 Supporting Information). The titration curves were fitted using a simple one-step binding model, as described in S1 Supporting Information to obtain the dissociation constant, *K*_*D*_, as discussed in Mitra *et al* [35]. The fitted *K*_*D*_ values are 283.6 ± 67.2 nM for GLP-1(7–37) and 178.1 ± 42.0 nM for Ex-4, each of which was averaged from measurements of three independently produced and purified GLP1R-ND batches. Ex-4 binds to GLP1R with similar but slightly higher affinity (1.59 ± 0.33 fold) than GLP-1, which agrees with previous studies using receptor expressing cells, semi-purified plasma membranes [51], and purified N-terminus extracellular domains of GLP1R [52, 53].

It is noted that 1–2 magnitude higher affinities were reported for the membrane-bound GLP1R enriched from disrupted eukaryotic cells by membrane preparation [54, 55]. To address the difference, we emphasize that the different approaches for quantitating GLP1R ligand binding may not be compared directly, especially since we are reporting the first purification of full-length GLP1R from mammalian expression system. Regardless, Schröder-Tittmann, *et al*. reported the *E*.*coli* recombinant expression and *in vitro* refolding of functional hGLP1R, where *K*_D_ value was unmeasurable for GLP-1 and determined to be ~180 nM for Ex-4 using fluorescence quenching and surface plasmon resonance methods [56], which is comparable to our results. On the other hand, a lower affinity can also be caused by (1) fluorescent labeling of the peptide ligands, (2) the non-native lipid environment and (3) absence of native receptor-affiliated proteins. First, the effect of the fluorescent labels was illustrated by performing cell-based cAMP assays to obtain the EC_50_s. The labeled GLP-1 and Ex-4 present EC_50_s of 1.18 ± 0.47 nM and 1.07 ± 0.11 nM, respectively (see S1 Supporting Information), about 2-order of magnitudes higher than the EC_50_s of non-labeled GLP-1 and Ex-4 (~10 pM range) [55, 57, 58]. Second, the POPC lipid bilayer that forms the nanodiscs is not expected to fully mimic a natural membrane environment. In fact, GLP1R was previously reported to localize in lipid raft with high cholesterol content, which could in turn affect the conformation and thereby the ligand affinity [59]. Finally, coupling to cognate G-proteins is known to enhance ligand affinities of GPCRs, which has been demonstrated in the pituitary adenylate cyclase-activating polypeptide receptor [60], parathyrpid hormone 1 receptor [37], somatostatin receptor [61] and the latrotoxin receptor [62]. In fact, such enhancement can result in more than 10-fold difference in ligand binding affinities of GPCRs [37, 60]. Future studies will be aimed at assembling nanodiscs with different lipid molecules to accommodate the receptor in a more nativelike environment and testing the affinities and receptor functions in the presence of receptor partners for formation of high-affinity ternary complexes as in the membrane environment.

### G protein activation by GLP1R-ND upon ligand binding

**Fig 8:**
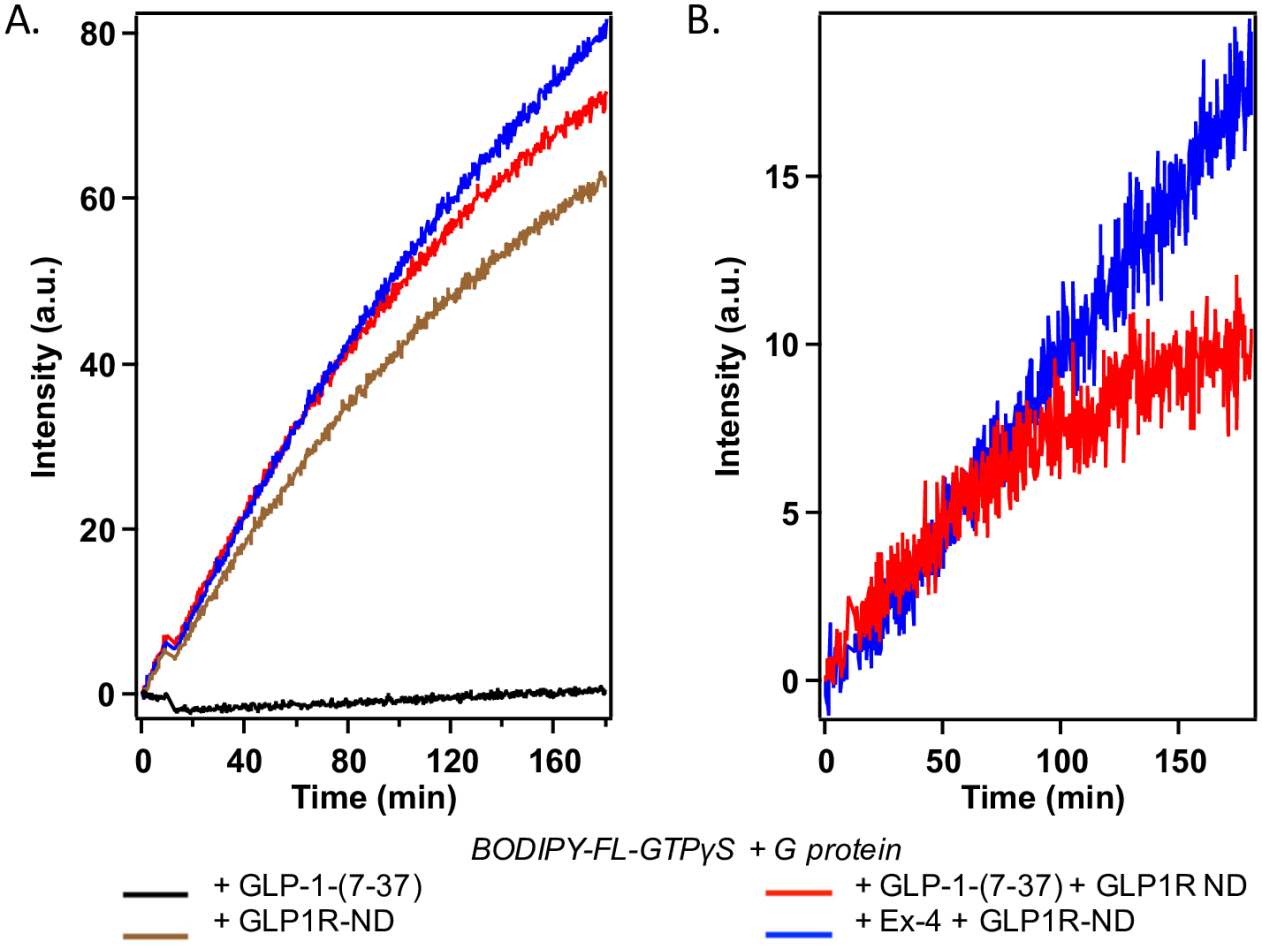
G -protein activity assay. (A) The fluorescence intensity monitored at excitation/emission 500 nm/512 nm with slit widths 2.5 nm/5 nm for the reaction mixture of BODIPY-FL-GTPγS and G_s_: addition of ligand GLP-1 alone shows no G_s_ activation (black), addition of GLP1R-ND alone shows an increase in fluorescence intensity, indicating the basal activity (brown), and addition of GLP1R-ND together with the GLP-1-(7–37) ligand (red) and addition of GLP1R-ND together with the Ex-4 ligand (blue) show an increase in intensity above basal level; (B) The activation of G_s_ by GLP1R-ND upon binding to GLP-1 and Ex-4 after subtraction of the basal activity.

We also used a fluorescence assay to examine the functionality of purified GLP1R-ND in G protein activation. The quenched fluorescence of free BODIPY-FL-GTPγS in solution is regained upon binding to G_s_. Thus the increase in fluorescence intensity indicates the activation of G_s_ upon the ligand binding of GLP1R-ND. Fig 8A presents a set of representative curves of three G protein activation experiments using three distinct preparations of purified receptor. The fluorescence intensity remains at low level in the absence of receptor, indicating that G_s_ was not activated and thus did not bind GTPγS. In the presence of purified receptor without ligand, the increase in fluorescence intensity shows the agonist-independent basal activity of GLP1R-ND. This could be explained by that many GPCRs have considerable basal activity that can be either increased or decreased by different classes of ligands [63, 64]. It is also likely that the relatively high basal activity is due to the absence of GDP in the current experimental setup. It has been reported that the addition of GDP is necessary to fill empty nucleotide binding sites of G protein, which can lower the basal GTPγS binding by as much as 10 folds [65, 66]. In addition, since GDP can compete with GTPγS for the binding site of G_α_, the presence of GDP also affect the agonist-stimulated response. Studies on the effect of GDP concentration on G protein activity assays for various GPCRs have been performed by several groups previously [66–69]. Regardless, Fig 8B clearly shows that upon the addition of agonist—GLP-1-(7–37) or Ex-4, the fluorescence signals increase compared to the basal activity, indicating further activation of G_s_ by GLP1R upon ligand binding. With the addition of Ex-4, the fluorescence intensity increases steadily at a constant rate over the 3h monitoring period, while the fluorescence intensity increase flattens out after 1.5h with the addition of GLP-1-(7–37), resulting in greater number of G proteins being activated with Ex-4. Such G protein activation results suggest Ex-4 has higher efficacy and is longer-acting in activating GLP1R, in agreement with previous studies [51–53, 70, 71]. The assays imply purified GLP1R-ND is functional in regard to activating G proteins.

## Discussion

In this study, we reported the first purification of full-length GLP1R from mammalian cells using the method of nanodiscs purification (Fig 3). The purified receptors in nanodiscs retained the capacity to bind ligands and activate G_s_, demonstrating the feasibility of the nanodisc purification method in the aspect of preserving biological functions of the receptors and thus having important implications in enabling future biophysical characterizations of GLP1R and potentially other family B GPCRs.

Obtaining purified GLP1R or other family B GPCRs in nanodiscs is expected to enable the studies on the receptors using various biophysical methods. Specifically, the purification of GLP1R in nanodiscs is capable of yielding quantities in the 10s of microgram amounts of functional receptor. Thus, in combination with labeling techniques, structural information of the receptors can be obtained using spectroscopic methods, such as Forster resonance energy transfer (FRET), resonance Raman, single-molecule spectroscopy, and even solution NMR for detecting conformational changes of isotopically (e.g., ^15^N) labeled hormone peptides upon binding to purified receptors [72–74]. Such labels can potentially be introduced to the GLP1R through unnatural amino acids mutagenesis and biorthogonal labeling [75–83], to other components of the nanodisc including MSPs and lipids, or to any agents that may interact with GLP1R-ND. Moreover, the current purification yield of ~0.25 μg per 10-cm dish of HEK293 cells can be further optimized, e.g. by screening for high expression and stable cell line or implementing bioreactors for growth of mammalian cultures in suspension [38, 39, 84], so as to enable spectroscopic studies including NMR characterizations that require large amount of protein samples. As a demonstration, our laboratory has successfully improved the purification yield of another family B GPCR, parathyroid hormone 1 receptor (PTH1R) from ~0.2 μg to ~1 μg per 10-cm dish of HEK293 cells.

Furthermore, the purification of receptors directly in nanodiscs would facilitate functional studies at the molecular level. In fact, GLP1R is the second family B GPCR that we successfully purified in nanodiscs [35], suggesting that the nanodisc purification can potentially be a general method to stably purify functional family B GPCRs. The purification method features the incorporation of GPCR targets into nanodiscs at the early stage of purification, minimizing the contact of GPCRs with detergents and thus stabilizing the proteins in lipid bilayer core, allowing functional purification [85]. Structural and functional studies of GPCRs will therefore benefit from the receptors being in a native-like lipid bilayer environment. For example, studies of GPCR interactions with downstream signaling proteins, including G protein and arrestin, are impossible using detergent-solubilized GPCRs, but can be achieved with GPCRs incorporated in nanodiscs [85, 86]. Moreover, the purification method allows controls of lipid compositions during the step of nanodisc assembling, thus eliminating additional procedures for reconstitution, where the transmembrane receptors in contact with detergent can be susceptible to denaturation and loss of functions. Since lipid-protein interactions are of great influence on the activity of GPCRs[87–90], the purification method would also allow the design of experiments to evaluate lipid effects on GPCR signaling.

In conclusion, we have used nanodisc purification method to purify GLP1R expressed in mammalian cells, and obtained purified receptors with preserved functions of ligand binding and G protein activity. This provides a useful tool to enrich functional GLP1Rs in a native-like environment for biophysical, biochemical and functional studies of the proteins without interference from other cell components. We propose that the nanodiscs purification method has the potential to be applied not only to other family B GPCRs, but also to GPCRs in other families and even other classes of membrane proteins in general.

## Supporting information

S1 Supporting Information (DOCX)
