# Supplementary material for: Purification of family B G protein-coupled receptors using nanodiscs: Application to human glucagon-like peptide-1 receptor
Source: PLoS One. 2017 Jun 13;12(6):e0179568. doi: 10.1371/journal.pone.0179568 (PMC5469476; doi:10.1371/journal.pone.0179568)
Supplement: S1 Supporting Information — (DOCX) [file pone.0179568.s001.docx]

**S1 Supporting information**

# ***Distribution of target protein into nanodiscs***

We previously spent extensive efforts optimizing the nanodisc assembly protocol by determining the membrane scaffold protein (MSP) to membrane protein ratio, the MSP to lipid ratio and the amount of Bio-Beads to be used in the nanodisc self-assembling step to achieve the incorporation of one GPCR per disc. Currently, in an example preparation, the concentration of membrane protein after solubilization is 180 μM, calculated using an average molecular weight of 40 kDa. To maintain the optimized ratio for nanodisc formation, (1 membrane protein: 8 MSP: 708 POPC), the assembly mixture contains 90 μM of MSP and 11.3 μM of membrane protein. Assuming each nanodisc contains 2 MSP molecules, 45 μM of nanodiscs will be formed. With the 11.3 μM of membrane protein, the ratio of formed nanodiscs to membrane protein is 4 nanodiscs: 1 membrane protein. Poisson distribution can be used to further calculate the probability of incorporating zero, one or two membrane proteins into a single nanodisc:

$P\left( x;\mu\right)={(e}^{-\mu}){(\mu}^{x})/x!$

where x is the number of proteins incorporated into one nanodisc and μ is the probability of incorporating 1 membrane protein in 1 nanodisc. Given the ratio of 4 nanodiscs: 1 membrane protein, μ = 0.25. Thus, the probability of a nanodisc incorporating 0 membrane protein is 77.9%, 1 membrane protein is 19.5% and 2 membrane proteins is 2.4%. The 2.4% of nanodiscs that may contain 2 membrane proteins is unlikely to affect our results or conclusions. Further, such 2.4% of nanodisc can contain any type of 2 membrane proteins, the probability of nanodiscs containing two target proteins (GLP1R) is even lower. In fact, ratio of overexpressed GLP1R to total membrane protein was determined to be around 1:1000 (0.1%) based on the expression level of the stable cell line. The ratio of nanodiscs containing two receptors to the ones containing one receptor is about 1:8100 (2.4% × 0.1% × 0.1% : 19.5% × 0.1%). Thus, the purified sample contains less than 0.02% of nanodiscs containing two receptors.

# ***GLP1R-ND particle size determined using transmission electron microscopy (TEM)***

We analyzed the size of the GLP1R-ND particles using ImageJ. Fig A shows the percentage frequency versus particle diameter histograms determined from four distinct TEM images that were obtained using three independently prepared GLP1R-ND batches. Each histogram was fitted into a Gaussian function (black curve) to examine the size distribution and the results are summarized in Table A. The averaged mean diameter for GLP1R-ND is 19.0 ± 1.6 nm and is reported in the main text. Overall, all four samples present satisfying homogeneity and dispersity as often observed for protein incorporated-nanodisc samples.

**Fig A. Histograms showing the particle size distributions of GLP1R-ND.**

**Table A.** **Mean particle diameters and standard deviations derived from the TEM-derived histograms.**

| **Sample** | **Mean diameter, *a*_0_ (nm)** | **Deviation, *σ* (nm)** | **Dispersity, *σ*_eff_ = *σ*/*a*_0_ (%)** |
| --- | --- | --- | --- |
| 1 | 18.1 | 2.8 | 15.7 |
| 2 | 19.2 | 2.7 | 14.1 |
| 3 | 19.5 | 3.3 | 16.9 |
| 4 | 19.3 | 4.1 | 18.1 |

# ***Data analysis of fluorescence anisotropy assay***

Fluorescence anisotropy experiments measured the binding affinity between GLP1R-ND and the peptide ligands, GLP-1-(7-37)-FAM and Ex-4-FAM. Each of the peptides was titrated with GLP1R-ND (0-400nM) for three times using three independently produced and purified receptor batches. The binding was fitted into a simple ligand-receptor binding model:

where *R* is a receptor, *L* is a ligand, *K_A_* and *K_D_* are association and dissociation constants, respectively. The solution of the model (Eq 2), as derived in Mitra *et. al*. [1], was used to fit the titration curves obtained by fluorescence anisotropy measurements to yield the *K_D_*.

where *r* is the measured anisotropy value; *C_ND_* is the concentration of GLP1R-ND; *C_P_* is the concentration of the peptide kept at a constant; *r_b_* is the anisotropy value of peptide bound to GLP1R-ND; and *r_f_* is the anisotropy value of the free peptide. *K_D_*, *r_b_*, and *r_f_* are the three fitting parameters for analyzing the titration curves.

The fitting results for each titration are summarized in Table B. In these anisotropy measurements, GLP1R-ND-bound peptides tumble at a much slower rate than the free ones, resulting in an increasing anisotropy value upon binding. Since the size of molecular entities and photochemical properties of the fluorescence probe determine anisotropy values, the anisotropy of the same free peptide (*r_f_)* shall remain the same throughout different batches of measurements, as is the anisotropy of the GLP1R-ND-bound peptide. With our experimental set-up, *r_f_* is directly observed in each titration at zero receptor concentration, and such *r_f_* values each averaged from three independent measurements were 0.0713 ± 0.0002 and 0.0817 ± 0.002 for GLP-1-(7-37)-FAM and Ex-4-FAM, respectively. Thus, these values were compared to the fitted *r_f_* values, 0.0712 ± 0.0031 for GLP-1-(7-37)-FAM and 0.0806 ± 0.0036 for Ex-4-FAM. Overall, the reproducibility of the titrations and fittings is satisfying, as indicated by the reproducible fitted *K_D_* and *r_b_* values.

**Table B. Fitted parameters for each titration curve of ligand binding assays.**

|  |  | | ***K_D_*** | ***r_f_*** | ***r_b_*** | ***K_D,_*_ave_** | ***r_f_*_,ave_** | ***r_b_*_,ave_** |
| --- | --- | --- | --- | --- | --- | --- | --- | --- |
| **GLP-1-(7-37)** | | **1** | 218.3 ± 39.4 | 0.0715 ± 0.0024 | 0.290 ± 0.016 | 283.6  ±  67.2 | 0.0712  ±  0.0031 | 0.304  ±  0.026 |
|  |  | **2** | 346.4 ± 42.4 | 0.0697 ± 0.0012 | 0.310 ± 0.014 |  |  |  |
|  |  | **3** | 286.0 ± 34.2 | 0.0723 ± 0.0014 | 0.311± 0.013 |  |  |  |
| **Ex-4** | | **1** | 177.9 ± 21.4 | 0.0830 ± 0.0019 | 0.308 ± 0.010 | 178.1  ±  42.0 | 0.0806  ±  0.0037 | 0.301  ±  0.019 |
|  |  | **2** | 178.8 ± 32.5 | 0.0812 ± 0.0028 | 0.297 ± 0.015 |  |  |  |
|  |  | **3** | 177.5 ± 15.9 | 0.0777 ± 0.0014 | 0.300 ± 0.007 |  |  |  |

Furthermore, we titrated both GLP-1-(7-37)-FAM and Ex-4-FAM with nanodiscs containing no receptor (empty-ND) as controls (Fig 7). These controls show much lower increase in anisotropy than the titrations with receptor containing nanodiscs (GLP1R-ND), but the titrations of empty-ND show slight increases in anisotropy for both Ex-4 and GLP-1-(7-37) with the increase relatively higher for GLP-1-(7-37). The controls imply that the ligand binding is specific to the receptor, but that GLP-1-(7-37) has a greater tendency to engage in non-specific binding to empty-ND. In fact, sequence analyses of GLP-1-(7-37) and Ex-4 (Table C) suggest that GLP-1-(7-37) is more hydrophobic. In addition, GLP-1-(7-37) is a neutral peptide (overall net charge of 0), while Ex-4 is acidic (3 negative charges). Both higher hydrophobicity and zero electrostatic charge can result in GLP-1-(7-37) with higher partition in lipid phase than in the aqueous phase relative to Ex-4, leading to the stronger non-specific interactions of GLP-1-(7-37) with empty-ND.

**Table C. Peptide hydrophobicity/hydrophilicity analysis.**

|  | **Peptide Sequence**  **Green:** hydrophobic  **Red:** acidic residues  **Blue:** basic residues | **Hydrophobicity /Hydrophilicity** | **Attribute** | **Grand average of hydropathicity (GRAVY)** |
| --- | --- | --- | --- | --- |
| **GLP-1-(7-37)** | **HAEGTFTSDVSSYLEGQAAKEFIAWLVKGRG** | Hydrophobic: 38.71% | Neutural | -0.235 |
|  |  | Acidic: 12.9% |  |  |
|  |  | Basic: 12.9% |  |  |
| **Ex-4** | **HGEGTFTSDLSKQMEEEAVRLFIEWLKNGGPSSGAPPPS** | Hydrophobic: 38.46% | **Acidic** | -0.692 |
|  |  | Acidic: 15.38% |  |  |
|  |  | Basic: 10.26% |  |  |

# ***Cell-based assay to determine potency of FAM-labeled GLP-1 and Ex-4***

The potency of FAM-labeled GLP-1 and Ex-4 was determined in a cell-based cAMP enzyme-linked immunosorbent assay (ELISA) using HEK293S cells stably transfected with the GLP1R gene. The GLP1R expressing cells were grown in 48-well plated, maintained under 5% CO2/ 95% air in 1:1 DMEM/F12 supplemented with 10% FBS, and induced with 0.55 mg/mL sodium butyrate and 2 µg/mL tetracycline. After ~40h of induction for receptor overexpression, the cells in each well were treated with 240 µL cAMP assay buffer (DMEM containing 100 µM IBMX, 2 mg/mL BSA, 35 mM HEPES, pH 7.4). Afterwards, 120 µL of binding buffer (50 mM Tris-HCl, pH 7.4, 100 mM NaCl, 5 mM KCl, 2 mM CaCl_2_, 0.5% FBS and 5% heat-inactivated FBS) containing the peptides at concentrations ranging from 100 to 0.001 nM was added, and the cells were stimulated for 10 min and then lysed with the lysis buffer (0.1 M HCl and 0.5% Triton X-100). The cAMP levels were quantified using direct cAMP ELISA kit (Enzo Life Science) according to the accompanying protocol. FAM-labeled GLP-1 induces intracellular cAMP accumulation with an EC_50_ of 1.18 ± 0.47 nM (Fig B). The Ex-4 displays an EC_50_ of 1.07 ± 0.11 nM. The EC_50_s of labeled GLP-1 and Ex-4 are roughly 2-order of magnitudes higher than the EC50s of non-labeled GLP-1 and Ex-4 (~10 pM range) [2-4], suggesting the effect of the fluorescence label.

**Fig B. cAMP production induced by FAM labeled GLP-1 and Ex-4.** Error shows average SD (n = 2).

**Reference**

1. Mitra N, Liu Y, Liu J, Serebryany E, Mooney V, DeVree BT, et al. Calcium-dependent ligand binding and G-protein signaling of family B GPCR parathyroid hormone 1 receptor purified in nanodiscs. ACS chemical biology. 2013;8(3):617-25.

2. Bueno AB, Showalter AD, Wainscott DB, Stutsman C, Marin A, Ficorilli J, et al. Positive Allosteric Modulation of the Glucagon-like Peptide-1 Receptor by Diverse Electrophiles. The Journal of biological chemistry. 2016;291(20):10700-15.

3. Thorens B, Porret A, Buhler L, Deng SP, Morel P, Widmann C. Cloning and functional expression of the human islet GLP-1 receptor. Demonstration that exendin-4 is an agonist and exendin-(9-39) an antagonist of the receptor. Diabetes. 1993;42(11):1678-82.

4. Gromada J, Rorsman P, Dissing S, Wulff BS. Stimulation of cloned human glucagon-like peptide 1 receptor expressed in HEK 293 cells induces cAMP-dependent activation of calcium-induced calcium release. FEBS Lett. 1995;373(2):182-6.
